# Symptom Severity, Body Image Dissatisfaction, and Movement Behaviors in Irritable Bowel Syndrome: Analysis of Quality of Life Determinants

**DOI:** 10.3390/healthcare14060714

**Published:** 2026-03-11

**Authors:** María Ángeles López-González, Eduardo José Fernández-Ozcorta, Félix Arbinaga, Manuel J. Arrayás-Grajera, Inmaculada Tornero-Quiñones

**Affiliations:** 1 Department of Integrated Teaching Methodologies, Faculty of Education, Psychology and Sports Science, University of Huelva, 21007 Huelva, Spain; mariadelosangeles.lopez1@alu.uhu.es (M.Á.L.-G.); marrayas@ddi.uhu.es (M.J.A.-G.); 2 EMOTION Research Group, University of Huelva, 21007 Huelva, Spain; 3 Department of Clinical and Experimental Psychology, University of Huelva, 21007 Huelva, Spain; felix.arbinaga@dpsi.uhu.es; 4 Physical Activity, Promotion of Values and Education Research Group, University of Huelva, 21007 Huelva, Spain; 5 Physical Activity, Health Promotion and Quality of Life (PAHPQoL) Research Group, University of Huelva, 21007 Huelva, Spain

**Keywords:** body image, cross-sectional studies, irritable bowel syndrome, physical activity, quality of life, severity of illness index

## Abstract

**Background:** Irritable Bowel Syndrome (IBS) exerts a profound burden on Health-Related Quality of Life (HRQoL) and psychosocial well-being. While lifestyle changes are recommended, the dose–response relationship between physical activity (PA) intensities, symptom severity, and body image remains unclear. This study analyzed the interrelationships between PA intensities, symptom severity, body image satisfaction, and HRQoL in IBS patients. **Methods:** A cross-sectional study was conducted with 40 adult patients (60% female; 32.53 ± 12.54 years) diagnosed via Rome III/IV criteria. Validated instruments were used to assess PA (IPAQ-SF), sedentary behavior (SBQ), HRQoL (IBS-QoL), symptom severity (IBS-SSS), and body image (BIS). Data were analyzed using Quantile Regression, Robust Linear Regression, and Causal Mediation Analysis. **Results:** Participants reported moderate symptom severity (210.1 ± 79.2) and high sedentary time (511.1 ± 265.0 min/day). Quantile Regression showed no statistically significant associations between PA intensities and clinical severity (all *p* ≥ 0.289). PA did not moderate the negative relationship between pain and HRQoL (*p* = 0.738). However, symptom severity was a significant predictor of body dissatisfaction (β = 0.36, *p* < 0.001). A sexual dimorphism was observed, as women exhibited higher baseline dissatisfaction and greater sensitivity to symptom worsening than men (β = −0.50, *p* = 0.004). **Conclusions:** Symptom severity is strongly associated with body dissatisfaction in IBS, particularly among women, independent of nutritional status. While PA did not directly mitigate symptoms in this cohort, the significant relationship with body image underscores the need for clinical interventions to integrate psychosocial support to address perceptual vulnerability.

## 1. Introduction

Irritable Bowel Syndrome (IBS) represents one of the most prevalent functional gastrointestinal disorders; moreover, a recent meta-analysis has indicated that global prevalence remains significant despite shifts in diagnostic criteria from Rome III to Rome IV [1], and it significantly compromises patients’ daily functioning [2]. IBS is a prevalent functional gastrointestinal disorder characterized by chronic abdominal pain, bloating and altered bowel habits [3,4]. IBS affects 3–5% of the world’s population, depending on the diagnostic criteria used [5,6].

The disease’s exact cause is not entirely clarified, but it is characterized by a complex interplay of comorbidities such as gastrointestinal disorders [7] and psychiatrics [8], immune system dysregulation [9], as well as lifestyle behaviors including physical exercise [10]. Moreover, it has been observed that diet can trigger symptoms in the vast majority of patients with IBS and that certain foods can worsen symptomatology [11]. Certain patients experience an amelioration of their symptoms by restricting certain foods and modifying their eating habits [12]. Over 60% of people with IBS report that their symptoms worsen several minutes after eating [13]. Nutritional management and lifestyle modifications constitute cornerstones in therapeutic approach. Implementing changes in this area can have positive effects on improving quality of life. The relationship between diet and progression of IBS leads to a reduction in certain foods that people usually consume [14].

Given that there are few effective treatment options available to manage IBS, many people experience significant deterioration in various areas of their lives. The uncertainty of the symptoms of this disease can cause considerable anxiety and shame, leading to social isolation [15]. IBS manifests as a biopsychosocial condition. These factors may include anxiety, depression, and uncomfortable situations that trigger more severe episodes of the disease [16]. Shame, in particular, is characterized by a profoundly distressing emotion due to its association with the perception of being inferior, unattractive or unworthy, and includes self-criticism [17].

IBS is associated with a significant deterioration in several dimensions of Health-Related Quality of Life (HRQoL). In fact, the HRQoL of people with IBS is worse than that of the general population, particularly in terms of energy/fatigue, functional limitations, bodily pain, and overall perception of health [18]. This can be caused by differences in the type, intensity, or duration of exercise performed among studies [19].

Regarding HRQoL, scientific evidence points to a sedentary lifestyle being associated with a higher probability of suffering from IBS [20,21]. Regular physical activity and exercise can be a lifestyle approach to reducing low-grade systemic inflammation [22], thereby reducing the risk of chronic diseases [23]. Increased physical activity has been shown to improve quality of life and reduce symptom severity, fatigue, anxiety, depression, abdominal pain, and the number of visits to doctors and nutritionists [24,25,26,27]. The results also suggest an inverse relationship between physical activity and the risk of gastrointestinal diseases [28]. The exercise interventions in this review were yoga [29,30], walking/aerobic physical activity, Tai Chi, mountain climbing, and Abaduajin Qigong activity [31]. However, the scientific literature does not provide specific details on the type and intensity of physical exercise [19]; in this regard, Lenover and Shenk [32] examined the relationship between physical activity intensity and increased risk of IBS in a sample of 921 individuals. Exercise intensity (sedentary/light) was significantly associated with an increased risk of IBS. Riezzo et al. [33] concluded that the intensity of intervention programs and their effects on the quality of the life of the people with IBS depended on whether the training was personalized or not. Therefore, further research is needed to understand how exercise affects this condition.

On the other hand, it is known that the symptoms of IBS and its treatment can increase stigma, linking it to feelings of shame commonly experienced by people with IBS [34] and with the impact of dissatisfaction with body image on most patients [35,36]. Recent research, including studies by Alleva and Tylka [37], has increasingly focused on the impact of chronic illness and pain on body image, revealing complex interactions between physical health and body perception. However, although body image deterioration has significant implications for the patient’s quality of life and progress, the underlying mechanisms have not been clearly explored. The study by de Trindade et al. [38] contributes important findings to the literature on IBS in relation to the mechanisms that could explain the deterioration of patients’ body image, as it suggests that greater IBS symptomatology is linked to greater dissatisfaction with body image.

On the other hand, age may be associated with lower levels of body dissatisfaction, which could also be explained by mechanisms of shame, which may be dimmed in older people. Likewise, women with IBS may experience greater impairments in their Health-Related Quality of Life compared to men, either on a physiological or psychosocial level [39]. Several common concerns among patients with IBS may reflect society’s expectations of women [40]. In general, it has been observed that patients diagnosed with IBS have a less favorable body image, they frequently experience negative bodily impressions, and they often perceive their body as a trigger for negative emotions, with minor appreciation of the body and self-criticism [41,42].

These alterations often occur as patients adapt and adjust to functional alterations or physical disfigurements, which can lead to a perception that their body is failing them, both in populations with chronic diseases [43] and in athletic populations [44]. IBS is a chronic gastrointestinal disorder that can be associated with a number of disabling characteristics, and it is known that the symptoms of the disease and their severity can negatively influence body image in chronic disease states [45]. In addition, many patients with IBS become ill during adolescence and early adulthood, when most people already have concerns about body shape, weight, physical function, self-esteem, and intimate relationships. The study by Muller et al. [46] indicates that body image is a concern for many patients with IBS, with two-thirds of the participants reporting that IBS had damaged their body image. Despite this data, none of the studies have attempted to evaluate body image or its comorbidities in patients with IBS.

Overall, greater physical activity may be associated with more positive psychological resources in people with IBS. However, the previous literature is limited because the different dimensions of physical activity, body image, and HRQoL in this population have not been thoroughly investigated. In this regard, the overall objective was to analyze the interrelationship between body satisfaction, movement behaviors, and the relationship with IBS. Specifically, we set out to (i) analyze the dose–response associations between physical activity intensities and severity; (ii) evaluate the moderating role of physical activity between pain and quality of life; (iii) determine the relationship between severity and body image; and (iv) examine the mediation model of body image on quality of life.

## 2. Materials and Methods

### 2.1. Study Design

A cross-sectional, observational, and correlational design was employed to elucidate the associations between physical activity, symptom severity, body image, and quality of life in patients with IBS.

### 2.2. Sample and Data Collection

The sample for this study consisted of 40 adult participants (n = 40) over the age of 18, with a mean age of 32.53 years (SD = 12.54). The gender distribution was 60% female (n = 24) and 40% male (n = 16). To ensure the clinical relevance of the sample, participants were recruited through a collaborative outreach strategy involving local and national patient support groups and specialized gastrointestinal health networks. These organizations facilitated the dissemination of the study protocol among their registered members via official internal communication channels. Following the initial expression of interest, a screening process was conducted to verify medical history and symptom persistence. Subsequently, a non-probabilistic convenience sampling method was used to select the subjects, who had a clinical diagnosis of IBS according to the Rome III and IV criteria, ensuring the homogeneity of the clinical sample. Participants were initially screened based on Rome III history; however, to ensure the sample’s clinical relevance and contemporaneity, all participants were re-evaluated, and only those who strictly met the Rome IV criteria were included in the final analysis.

From a clinical standpoint, the diagnosis was based on the updated Rome IV criteria (2016), which define IBS as recurrent abdominal pain at least one day per week for the past three months. This pain must be associated with two or more of the following factors: (1) relationship with defecation, (2) association with a change in stool frequency, and (3) association with a change in stool shape or appearance [47]. This version introduced significant changes from the Rome III criteria; the term “discomfort” was removed from the main definition and the phrase “improvement with defecation” was changed to “related to defecation,” recognizing that defecation does not always relieve symptoms [46]. Likewise, the word “onset” was removed when referring to changes in stool frequency or form to focus on their temporal association with abdominal pain [47].

### 2.3. Instruments

The following validated instruments were administered to characterize the sample and evaluate clinical and psychosocial variables:

- Sociodemographic and Anthropometric Data: An initial questionnaire designed ad hoc was administered to collect information on age, sex, and anthropometric parameters such as weight and height, used to calculate Body Mass Index (BMI) scores. For the analyses, nutritional status was dichotomized, considering participants with a BMI of 25 kg/m^2^ or more as “overweight/obesity” (which includes both overweight and obesity categories).
- Physical Activity: Physical activity level was measured using the short version of the International Physical Activity Questionnaire (IPAQ-SF). This 7-item instrument assesses the intensity (light, moderate, and vigorous), frequency, and duration of physical activity performed in the last week. Results are expressed in metabolic equivalents (METs-min/week).
- Sedentary Behavior: The Sedentary Behavior Questionnaire (SBQ) [48] was used, incorporating the two additional items from the Spanish validation (eating while sitting and resting while lying down). The Spanish version of this instrument has demonstrated good-to-excellent test–retest reliability (ICC = 0.75–0.90). The questionnaire assesses the time spent on 11 sedentary activities on both weekdays and weekends, with response options ranging from “none” to “six hours or more.”
- Quality of Life: Irritable Bowel Syndrome Quality of Life Questionnaire (IBS-QoL) was used. The 34-item version validated in Spanish [49] was used, demonstrating excellent internal consistency (Cronbach’s α = 0.95). This instrument assesses the impact of IBS on overall well-being using a 5-point Likert scale (1: never to 5: always). Higher scores on this instrument reflect a better perceived quality of life.
- Symptom Severity: The severity of the syndrome was assessed using Irritable Bowel Syndrome Severity Score (IBS-SSS) [50], in its Spanish-validated version [51], which has demonstrated robust internal consistency (Cronbach’s α = 0.81). It consists of five items that measure the intensity and frequency of abdominal pain, bloating, dissatisfaction with bowel habits, and the impact of IBS on daily life over the last 10 days. The total score (0–500) allows severity to be classified as mild (75–175), moderate (175–300), or severe (>300).
- Body Image: The Body Image Scale (BIS) [52], validated in Spanish by Gómez-Campelo et al. [53], was applied, demonstrating an outstanding internal consistency (Cronbach’s α = 0.93). It is a 10-item scale that briefly assesses the affective, behavioral, and cognitive dimensions of body image. It uses a 4-point response scale (0: not at all to 3: very much), where higher scores indicate greater distress or dissatisfaction with body image.

### 2.4. Ethical Considerations

This study was conducted in accordance with the guidelines of the Declaration of Helsinki. The project was submitted to the Research Ethics Committee. The committee granted its approval with internal reference number 2025.310, recorded in minutes 12/2025 dated 18 July 2025.

During the preparation of this manuscript and study, the authors used Gemini (Google) to assist in the interpretation of complex statistical analyses, the structuring of the scientific narrative, and the technical editing of the text. The authors have thoroughly reviewed and edited the generated content and assume full responsibility for the integrity and accuracy of the results and conclusions presented in this publication.

### 2.5. Statistical Analyses

First, outlier detection and data quality assessment were performed. To address missing data (overall missingness: 12.77% across the dataset), Multiple Imputation by Chained Equations (MICE) was employed using the mice package [54], thereby ensuring the robustness of subsequent estimations and preventing biases associated with listwise deletion. Following methodological recommendations for this missingness rate under a Missing at Random (MAR) assumption, m = 10 multiply imputed datasets were generated to sufficiently minimize sampling variability. The imputation procedure incorporated all targeted variables (sociodemographic characteristics, symptom severity, body satisfaction, physical activity, and quality of life dimensions) to preserve the underlying correlational structure. Finally, statistical analyses were conducted across all 10 datasets, and findings were pooled into single estimates using Rubin’s rules.

Second, a descriptive analysis was conducted to characterize the sample (n = 40), utilizing means and standard deviations for normally distributed variables and frequencies for categorical ones. Since Shapiro–Wilk tests revealed non-normal distributions for physical activity and pain variables (*p* < 0.05), both parametric and non-parametric statistics (median and interquartile range) are reported. Subsequently, Spearman’s correlation matrix (rho) was calculated to examine the relationships between the study variables.

Third, advanced analytical models were executed to address the study objectives. A competitive model comparison was performed using Quantile Regression (tau = 0.5) to identify the physical activity intensity with the greatest predictive capacity for median severity. The Akaike Information Criterion (AIC) was used for the selection of the most parsimonious model, with standard errors estimated via bootstrapping with 1000 replicates.

The relationship between severity and body dissatisfaction was analyzed using Robust Linear Regression with the MM-estimator to mitigate the influence of potential outliers. This model was adjusted for sex, age, and BMI, employing robust standard errors (HC3) to protect estimates against heteroscedasticity. To evaluate the buffering effect of physical activity on quality of life, a moderation model was employed. In this model, pain intensity and total MET variables were mean-centered, and inference was similarly based on HC3 robust standard errors.

Finally, a Causal Mediation Analysis was conducted to determine whether the BIS appears to act as a mediating factor in the relationship between symptom severity and quality of life. To validate the significance of the indirect effect (ACME) and the direct effect (ADE), a non-parametric bootstrapping procedure with 1000 simulations was employed. The statistical significance level was set at *p* < 0.05 for all tests. Data processing and statistical analyses were performed using R software, version 4.5.1.

## 3. Results

### 3.1. Biopsychosocial Profile, Physical Activity, and Sedentary Patterns

#### 3.1.1. Sample Description

The final sample consisted of 40 participants (n = 40). Demographic and clinical dimensions were analyzed, alongside patient-reported outcomes. Regarding the sociodemographic profile (see Table 1), the sample was predominantly female (60%, n = 24). The mean age was 32.53 years (SD = 12.54), with a range from 18 to 67 years.

Concerning anthropometric parameters, both height (M = 165.9 cm, SD = 8.8) and weight (M = 73.6 kg, SD = 15.5) followed normal distributions (*p* > 0.05). Nevertheless, BMI revealed a trend toward excess body weight (M = 26.8 kg/m^2^, SD = 5.5). Nutritional status classification indicated that, while 45% of the participants were within the normal weight range (n = 18), more than half of the sample (52.5%, n = 21) presented with overweight or obesity.

Total physical activity volume, measured via IPAQ, reached a mean of 2503.9 MET-min/week (SD = 1860.1). The distribution of physical activity levels revealed that 50% of the participants maintained a “High” level, 40% a “Moderate” level, and only 10% were classified as “Low.” Despite the reported metabolic volume, the mean sedentary time was notably elevated, at 511.1 min/day (SD = 265.0). Physical activity intensity variables (vigorous, moderate, and light) showed significant deviations from normality (*p* < 0.001), suggesting high inter-individual variability in the movement patterns of the sample.

Normality tests indicated that the two primary outcome variables followed a normal distribution. The IBS-SSS showed a mean IBS severity of 210.1 points (SD = 79.2, *p* = 0.324), placing it within the moderate severity range. Regarding the IBS-QoL, the mean score was 67.0 (SD = 19.9, *p* = 0.543). Conversely, the specific pain dimensions did not meet the assumption of normality. Both pain intensity (Mdn = 50.0, IQR = 25.0) and pain frequency (Mdn = 40.0, IQR = 32.5) exhibited skewed distributions (*p* < 0.01), indicating that a proportion of the sample experiences extreme pain levels that bias the arithmetic mean.

Finally, the BIS showed a significant deviation from normality (*p* = 0.042), with a mean score of 18.8 (SD = 6.9) within a range of 9 to 35 points. This suggests a distorted or heterogeneous perception of body image within this clinical population.

#### 3.1.2. Correlational Analysis

**Figure 1:**
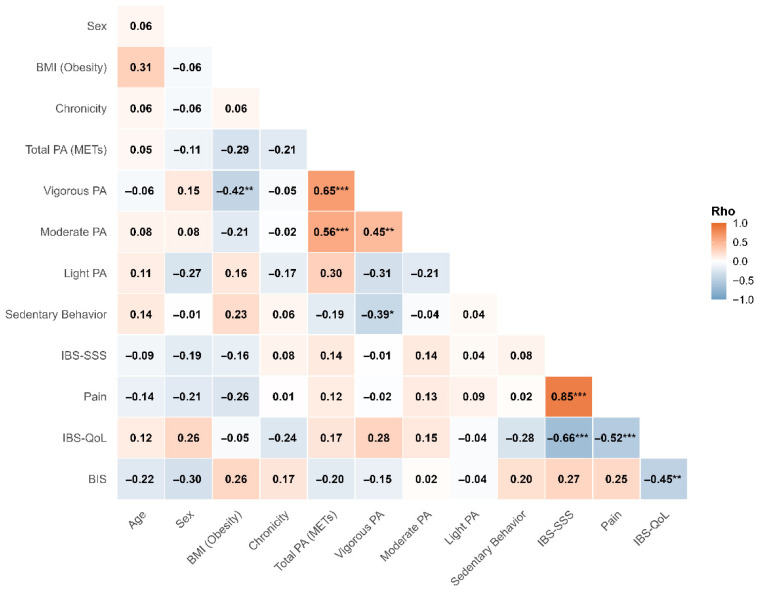
Spearman correlation matrix between sociodemographic, clinical, physical activity variables, and patient-reported outcomes. Note. * = *p* < 0.05; ** = *p* < 0.01; *** = *p* < 0.001. IBS-SSS = IBS Severity; IBS-QoL = Quality of Life; BIS = body dissatisfaction; METs = Total Energy Expenditure.

The correlational study of the variables revealed a large positive association between the IBS-SSS and pain intensity (rho = 0.85, *p* < 0.001), a finding that validates the internal consistency of the clinical instruments applied in the study (see Figure 1). Complementarily, the IBS-QoL exhibits a robust negative correlation with both severity (rho = −0.66) and perceived pain (rho = −0.52). These results confirm that symptom burden constitutes the primary detractor of perceived well-being in this population.

Furthermore, the weakness of the associations observed between the PA dimensions and the main clinical variables is methodologically relevant. Total energy expenditure, expressed in MET-min/week, as well as vigorous and moderate intensities, showed no significant correlations with syndrome severity or quality of life. Finally, the analysis of psychosocial vulnerability using the BIS showed a moderate negative association with the IBS-QoL (rho = −0.45) and a positive correlation with the IBS-SSS (rho = 0.27). Additionally, a negative association between sex and the BIS was identified (rho = −0.30).

### 3.2. Regression Analysis and Predictive Modeling

To identify which physical activity dimension (vigorous, moderate, or light) possesses the greatest predictive capacity for the IBS-SSS, a competitive model comparison was conducted using Quantile Regression (tau = 0.5).

The AIC evaluation revealed that the three models (light, moderate, and vigorous intensity) are statistically equivalent, as the differences in ΔAIC were less than 2 units. The model based on light intensity (walking) was identified as the most parsimonious (AIC = 470.5), although the evidence precludes conclusively prioritizing one intensity over another. Furthermore, a detailed analysis of robust coefficients enabled a comparison of the effect magnitude for each type of physical activity (see Table 2). Vigorous physical activity showed the largest relative effect magnitude (β = −0.38); however, this association did not reach statistical significance (*p* = 0.289) due to the high variance detected. Meanwhile, the effects of light and moderate intensities were statistically negligible (*p* > 0.40).

### 3.3. Evaluation of the Buffering Effect of Physical Activity

We evaluated whether total physical activity (MET-min/week) acts as a moderating factor capable of mitigating the negative relationship between pain and IBS-QoL (Table 3).

The analysis revealed that, although pain is a determining predictor of the reduction in quality of life, physical activity does not exert a significant moderating effect in the studied sample (*p* = 0.738). As detailed in Table 3, the main effect of pain showed a significant negative association with quality of life (β = −0.35, *p* = 0.002). Regardless of the volume of exercise performed, each one-point increase in pain intensity is associated with a 0.35-point decrease in the IBS-QoL scale. The direct effect of physical activity on quality of life did not reach statistical significance (β = −0.003, *p* = 0.068), and therefore, no definitive association can be established within this sample. The interaction (Pain × METs) was virtually null (*p* = 0.738), indicating that the relationship between pain and well-being is not altered by the patient’s PA level. Finally, sex did not show a significant influence on quality of life (β = 7.36, *p* = 0.237), indicating that the negative relationship between pain and perceived well-being is cross-sectional across both genders.

**Figure 2:**
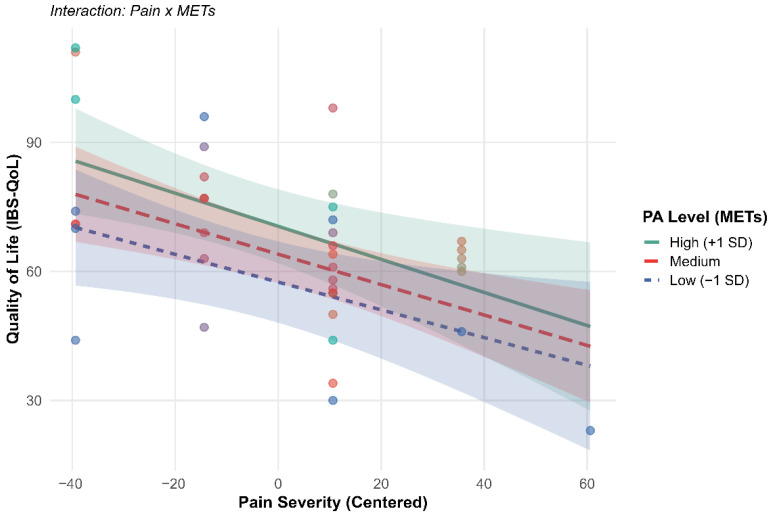
Effect of pain on QoL at different PA levels. Note. The parallel slopes confirm the absence of interaction. Color coding denotes physical activity levels based on METs: green indicates high physical activity, red represents moderate activity, and blue denotes low physical activity levels.

As observed in the simple slope analysis (Figure 2), the inverse relationship between pain and quality of life remains constant and independent of the patient’s exercise level. This indicates the absence of a direct biological protective effect of PA against the burden of pain symptoms.

### 3.4. Determinants of Body Image Dissatisfaction

To quantify the predictive value of IBS-SSS on the BIS, a Robust Linear Regression model was constructed. The model was adjusted for critical biological and demographic variables: sex, age, and nutritional status (presence of overweight or obesity) (Table 4).

The model revealed that symptom severity is strongly associated with body dissatisfaction (*p* < 0.001), after controlling for weight and gender. As detailed in Table 4, IBS severity showed a standardized coefficient (β = 0.36). Specifically, an increase of 50 points in the IBS-SSS score—representing the Minimum Clinically Important Difference (MCID)—was associated with a 1.5-point increase in body dissatisfaction. This indicates that even a minimum perceptible worsening of clinical symptoms correlates with a measurable decline in body image perception.

Regarding sexual dimorphism, this emerged as the predictor with the greatest relative weight in the model (β = −0.50, *p* = 0.004), indicating that men report systematically lower levels of dissatisfaction than women (this should be interpreted with caution due to the limited sub-sample size). Finally, overweight/obesity showed a positive and independent association with the BIS score (β = 0.33, *p* = 0.019), confirming the expected relationship between BMI and aesthetic self-perception. Conversely, age did not show a statistically significant influence on body dissatisfaction in this population (*p* = 0.559), suggesting that the perceptual vulnerability associated with IBS is cross-sectional across the different age groups in the sample.

**Figure 3:**
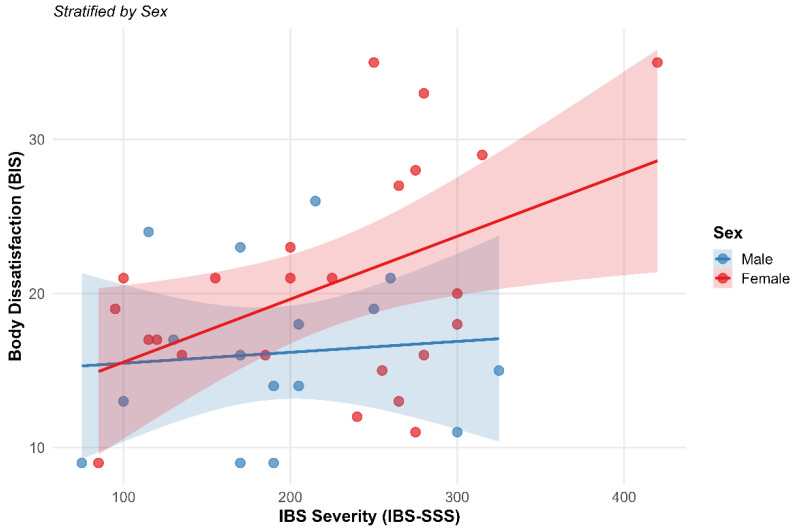
Relationship between symptom severity (IBS-SSS) and body dissatisfaction (BIS) stratified by sex. Note: Points represent individual observations (n = 40). Solid lines indicate the estimated linear regression trend for each group, while shaded areas represent the 95% confidence intervals. A positive correlation is observed in both groups, where an increase in symptom severity predicts a rise in body dissatisfaction—an effect that is intensified in the female group.

Figure 3 shows the relationship between IBS-SSS and the BIS stratified by sex. Robust regression analysis confirms that symptom severity is a statistically significant and positive predictor of the BIS score (β = 0.36, *p* < 0.001). The data indicate that patients with a severity score exceeding 300 points on the IBS-SSS present with higher levels of body dissatisfaction regardless of their nutritional status.

A sexual dimorphism is observed in both the intercept and the slope of the relationship. The male group reports lower baseline dissatisfaction (β = −0.50, *p* = 0.004) and a more moderate slope of increase compared to the female group. The female group begins with higher dissatisfaction levels and shows a more acute response to the worsening of clinical symptoms. At extreme severity levels, above 350 points, an increase in response variance and a widening of the 95% confidence intervals are observed, particularly within the female sample.

### 3.5. Mediation Analysis of Body Image Satisfaction

The mediating role of the BIS in the relationship between IBS-SSS and IBS-QoL was analyzed using a causal mediation procedure with non-parametric bootstrapping of 1000 simulations.

**Figure 4:**
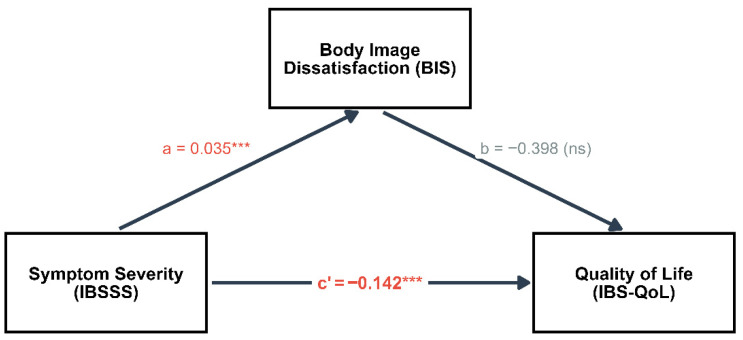
Causal mediation model of the indirect effects of symptoms on quality of life. *** The diagram displays standardized path coefficients. Statistical significance is indicated by asterisks (*p* < 0.001) while ns denotes paths that did not reach statistical significance (*p* > 0.05).

The results determined a statistically significant total effect (see Figure 4), indicating that for each unit of increase in the IBS-SSS, the IBS-QoL decreases by 0.155 points (95% CI [−0.213, −0.097], *p* < 0.001). The average direct effect (ADE) of symptoms on quality of life was significant (Estimate = −0.142, *p* < 0.001), whereas the average causal mediation effect (ACME) through the BIS did not reach statistical significance (Estimate = −0.014, 95% CI [−0.041, 0.009], *p* = 0.226). The proportion of the effect of symptoms on quality of life that appears to act through body image was quantified at 8.8%. In the path diagram coefficient analysis, severity showed a significant relationship with body image (path a: β = 0.035, *p* < 0.001), while the relationship between body dissatisfaction and quality of life remained non-significant (path b: β = −0.398, *p* = 0.072). The decomposition of effects via bootstrapping, detailed in Figure 4, shows that the confidence interval for the indirect effect crosses zero, in contrast to the negative shifts observed in the direct and total effects.

## 4. Discussion

The objectives of the research were to examine the relationship between physical activity levels and the severity of IBS symptoms, as well as to determine the moderating role of physical activity between pain and quality of life. Furthermore, the relationship of the disease with body image was studied, along with the mediating role of this variable in the quality of life of individuals with IBS.

Regarding the first objective—evaluating the dose–response associations between physical activity (PA) intensities and clinical severity—no statistically significant associations were found between PA intensity (light, moderate, or vigorous) and symptom severity. These findings contest conventional clinical guidelines that advocate for “more exercise” directly reducing IBS symptoms [24,25,32]. Systematic reviews [27,55] indicate that while PA is suggested to improve symptoms, the certainty of this evidence remains very low, complicating the confirmation of a real relationship and questioning standard clinical advice. This study indicates that while low-to-moderate intensity exercise is safe, the effects of high-intensity exercise on IBS symptoms remain inconsistent and do not show a direct, proportional improvement in severity [55].

Regarding the second objective—examining the moderating role of physical activity between pain and quality of life—the interaction term between pain and METs was found to be null (*p* = 0.738). Pain was a potent negative predictor of quality of life regardless of the exercise level (B = −0.35, *p* = 0.002). Physical activity does not function as a “buffer” or a biological resilience factor against the psychological impact of pain [27]. The authors conclude there is likely little or no difference between physical activity interventions and usual care concerning quality of life and abdominal pain. Liguori [56] concludes that while PA may improve global IBS symptoms, it is unclear whether it improves quality of life and abdominal pain compared to other non-PA interventions. A physically active patient experiences the same deterioration in their quality of life during a pain episode as a sedentary patient. This is crucial because it shifts the therapeutic focus: exercise may support baseline well-being, although this direct effect did not reach statistical significance (*p* = 0.068), but it does not “buffer” or “anesthetize” the functional consequences of acute IBS pain. Clinical research [27] reinforces that pain is the primary detractor of quality of life. Irrespective of whether the patient meets exercise recommendations (150–300 min/week) [57], pain severity potently and negatively predicts functional well-being.

Regarding the third objective, which examines the relationship between severity and body image, symptom severity emerged as a robust and independent predictor of body dissatisfaction (β = 0.36, *p* < 0.001). Biological sex (with women reporting higher dissatisfaction; this should be interpreted with caution due to the limited sub-sample size) and overweight/obesity were also key predictors. This constitutes one of the study’s most innovative findings, demonstrating that Irritable Bowel Syndrome (IBS) distorts body self-perception not only through aesthetic factors (BMI) but also through the symptom burden itself. Patients perceive their bodies as a “failing entity,” leading to a sense of disconnection and rejection of their physical image. Prior research [58] confirms that the severity of gastrointestinal symptoms is significantly associated with negative physical self-perception. In specific IBS populations [35,37,59], patients are characterized by diminished representations of body schema, body image, and bodily sense compared to healthy individuals [34], which is further linked to a lower quality of life [59]. The greater vulnerability observed in women suggests that aesthetic social pressure exacerbates the biological burden of the disease. It has been documented that women with IBS consistently report lower levels of quality of life and poorer body image compared to men [60]. Many women in Western countries are socialized to prioritize body image and thinness, which can often translate into increased anxiety regarding symptoms such as extreme bloating [40]

Finally, regarding the fourth objective, which examines body image as a mediator between symptom severity and quality of life, the relationship between symptoms and quality of life was found to be predominantly direct (ADE = −0.142, *p* < 0.001). This significant finding aligns with the study by Ake et al. [61], which identified a direct association between symptom severity and the deterioration of all quality-of-life dimensions. Mediation through body image (BIS) did not reach statistical significance (ACME = −0.014, *p* = 0.226), accounting for only 8.8% of the total effect. Although the disease is negatively related to body image, this impairment is not the primary mechanism through which patients experience a decline in quality of life.

Our results are consistent with Geller et al. [41], who explored how IBS relates to greater body dissatisfaction but suggested that factors such as self-criticism or specific anxiety often play a stronger mediating role in psychological distress than physical image itself. Similarly, Trindade et al. [62] utilized structural equation modeling to demonstrate that while severity contributes to HRQoL impairment, gastrointestinal anxiety tends to be a more potent mediator than aesthetic or physical self-concept factors. The impact of IBS appears to be “raw” and direct: pain and urgency disrupt daily life due to their inherent sensory and restrictive nature, rather than through a cognitive–psychological pathway that appears to act as a mediating factor in the association with physical self-concept. This reinforces the notion that IBS carries a biological burden so substantial that it bypasses typical psychological mediation mechanisms [63]. Furthermore, although Snijker et al. [64] focused on life satisfaction, their work highlights that physical and mental quality of life are independent determinants and that symptom severity exerts a weight of its own that is not always mediated by psychological burden.

The current study, although providing new evidence on the connection between symptom severity and body image in IBS, is constrained by methodological limitations that are a result of its design. The cross-sectional design allows for the identification of associations and interrelationships, but not for determining definitive causality. Advanced statistical models, including mediation and regression analysis, were employed to examine directionality; however, the findings should be viewed with caution due to the inability to establish the temporal sequence of the effects. A longitudinal study is needed to verify whether body dissatisfaction increases symptom perception over the long term. In relation to the sample size (n = 40), the complex statistical models presented (mediation and moderation) should be interpreted as exploratory. These findings provide a basis for future research but require validation in larger cohorts to ensure coefficient stability and higher statistical power. This might have led to the lack of statistical significance of the moderating effect of physical activity and the full effect in which body image appears to act as a mediating factor. The measurement of movement behaviors was based on self-report instruments such as the IPAQ-SF and the SBQ. These tools are vulnerable to memory bias and social desirability, which may result in an overestimation of total physical activity and complicate the accurate detection of a dose–response association. The coexistence of high reported physical activity and elevated sedentary time in our sample suggests a potential self-report bias. Therefore, the lack of observed associations between physical activity and clinical outcomes should be interpreted with caution, as it may reflect the instrument’s limited precision in capturing intensity rather than a definitive absence of biological effect.

Based on the current study’s limitations and significant findings, future research directions are proposed as follows: Future studies should incorporate longitudinal or interventional designs, such as randomized controlled trials, to conclusively verify the causality and directionality of observed relationships, a prerequisite for translating findings into clinical practice. Further research should intensify the examination of sexual dimorphism, investigating the psychosocial and biological factors that account for the aesthetic pressure and symptomatic load in female patients with Irritable Bowel Syndrome. Future research should utilize objective measures of movement patterns (e.g., accelerometers) to obtain more precise estimates of physical activity and sedentary periods, thereby validating the study’s findings on marginal impact and determining an ideal and safe exercise intensity. The evidence ultimately points to a need to develop and evaluate the effectiveness of multidisciplinary treatments that combine pharmacological or dietary pain management methods with a separate psychosocial component focused on rehabilitation of body image, physical self-perception, and reduction of shame in patients with high levels of symptomatic severity.

## 5. Conclusions

The results suggest that the therapeutic management of IBS could benefit from evolving from generic physical activity (PA) recommendations toward a multidisciplinary approach that integrates pain management and body image (BI) rehabilitation. Given that, in this specific cohort, PA did not show a direct relationship with symptom reduction or quality of life, prioritizing psychological interventions and the reduction of sedentary time may offer a more tailored strategy for improving patient prognosis. These findings highlight the need to address psychological mediators alongside lifestyle changes.

## Figures and Tables

**Table 1:** Descriptive analysis of study variables.

Variable	M (SD)/n (%)	Mdn (IQR)	Range	W (*p*)
Demographics				
Age (years)	32.53 (12.54)	29.00 (12.00)	18–67	<0.001
Sex (females)	24 (60%)	-	-	-
BMI	26.8 (5.5)	25.4 (9.3)	15.6–38.7	0.039
Chronicity (years)	6.75 (8.04)	5.00 (8.00)	1–44	<0.001
Physical Activity				
Vigorous METsa	976.0 (1359.1)	480.0 (1360.0)	0–6720	<0.001
Moderate METs	553.0 (752.6)	300.0 (680.0)	0–3360	<0.001
Light METsa	974.9 (913.4)	693.0 (1163.3)	0–2772	<0.001
Total METsl	2503.9 (1860.1)	2108.0 (2148.8)	278–10,196	<0.001
Sedentary Time (min/day)	511.1 (265.0)	493.9 (458.0)	96–1365	0.038
Patient-Reported Outcomes				
IBS-SSS	210.1 (79.2)	205.0 (117.5)	75–420	0.324
Pain	39.4 (26.5)	50 (50)	0–100	<0.001
IBS-QoL	67.0 (19.9)	66.5 (21.3)	23–112	0.543
BIS	18.8 (6.9)	17.5 (7.5)	9–35	0.042

Note. M = Mean; SD = Standard Deviation; Mdn = Median; IQR = Interquartile Range; W = Shapiro–Wilk; BMI = Body Mass Index; IBS-SSS = Irritable Bowel Syndrome Symptom Severity Score; BIS = Body Image Satisfaction; IBS-QoL = Irritable Bowel Syndrome Quality of Life; METs = Metabolic Equivalents. Note: Chronicity (years) refers to the duration of IBS symptoms since clinical diagnosis.

**Table 2:** Comparison of intensity predictors on median severity (tau = 0.5).

Model	*B*	*SE_boot_*	*β*	*t*	*p*	AIC	ΔAIC
Vigorous	−0.022	0.021	−0.38	−1.08	0.289	471.6	+1.10
Moderate	0.012	0.031	0.11	0.38	0.705	471.5	+0.94
Light	0.019	0.025	0.22	0.75	0.457	470.5	

Note. *B* = change in the IBS-SSS median per MET unit; *SE_boot_* = standard error obtained via 1000 bootstrap replicates; ΔAIC = difference relative to the model with the lowest AIC.

**Table 3:** Regression model for the moderation of quality of life (IBS-QoL) by physical activity.

Predictor	*B*	95% CI	*SE_HC_*_3_	*β*	*t*	*p*
(Intercept)	64.01	[56.82, 71.19]	3.54	18.09	<0.001	64.01
Pain (Centered)	−0.35	[−0.57, −0.13]	0.11	−3.28	0.002	−0.35
METs (Centered)	0.003	[−0.000, 0.007]	0.002	1.89	0.068	0.003
Interaction (Pain × METs)	−0.000	[−0.000, 0.000]	<0.001	−0.34	0.738	−0.000
Sex (Male)	7.36	[−5.07, 19.80]	6.12	1.20	0.237	7.36

Note. *B *= unstandardized coefficient; 95% CI = 95% confidence interval for *B*; *SE*_*HC*3_ = robust standard error (HC3 estimator); *β* = standardized coefficient; *p*-values are based on robust standard errors.

**Table 4:** Results of the robust linear regression model (MM-estimator) for the prediction of body dissatisfaction (BIS).

Predictor	*B*	95% CI	*SE_HC_*_3_	*β*	*t*	*p*
(Intercept)	12.33	[3.28, 21.38]	4.42	0.00	2.79	0.009
IBS-SSS	0.03	[0.01, 0.05]	<0.01	0.36	3.46	<0.001
Sex (Male)	−6.65	[−10.98, −2.31]	2.11	−0.50	−3.14	0.004
BMI (Overweight/Obesity)	4.36	[0.75, 7.97]	1.76	0.33	2.46	0.019
Years	0.05	[−0.12, 0.22]	0.08	0.09	0.59	0.559

Note. *B* = unstandardized coefficient; 95% CI = 95% confidence interval for *B*; *SE*_*HC*3_ = robust standard error (HC3 estimator); *β* = standardized coefficient; *p*-values are based on robust standard errors.

## Data Availability

The raw data supporting the conclusions of this article will be made available by the authors on request.

## Abbreviations

The following abbreviations are used in this manuscript:

IBS	Irritable Bowel Syndrome
HRQoL	Health-Related Quality of Life
IBS-QoL	Irritable Bowel Syndrome Quality of Life Questionnaire
IBS-SSS	Irritable Bowel Syndrome Symptom Severity Score
BIS	Body Image Scale
BMI	Body Mass Index
SBQ	Sedentary Behavior Questionnaire
ST	Sedentary Time
IPAQ-SF	International Physical Activity Questionnaire–Short Form
METs	Metabolic Equivalents
